# 

**Published:** 1858-09

**Authors:** 


### DENTAL REGISTER

### OF THE WEST.

EDITED BY

J. TAFT AND GEO. WATT.

September, 1858.

Published Quarterly at $3 Per Annum.

CINCINNATI:

J. TAFT, PUBLISHER AND PROPRIETOR.

1858.

